# Bromine as a Disinfectant

**Published:** 1891-10

**Authors:** 


### Bromine as a Disinfectant.

Bromine as a disinfectant is said to be coming to the front. It is an inexpensive by-product of the manufacturer of salt, selling at seventy cents a pound, and in solution containing one part in weight to about eight hundred of water; it may be used freely without affecting anything which it may touch. A few gallons used daily will remove all ammoniacal orders from the stables, or a few quarts will thoroughly deodorize the entire plumbing system of an ordinary house. The undilated bromine is strongly corrosive, and if it touches the skin causes a painful burn.—[*The Pacific Record.*

